# Polarization-independent split bull’s eye antennas for infrared nano-photodetectors

**DOI:** 10.1038/srep39106

**Published:** 2016-12-19

**Authors:** Meng Yang, Fang-Fang Ren, Lin Pu, Long Xiao, Yun Sheng, Junzhuan Wang, Hai Lu, Youdou Zheng, Yi Shi

**Affiliations:** 1 Key Laboratory of Advanced Photonic and Electronic Materials and School of Electronic Science and Engineering, Nanjing University, Nanjing 210093, China; 2 Collaborative Innovation Center of Advanced Microstructures, Nanjing University, Nanjing 210093, China

## Abstract

Split bull’s eye (SBE) antennas exhibit much larger extraordinary optical transmission and strong polarization dependence rather than bull’s eye (BE) antennas in the infrared range due to the introduced sub-wavelength slit. Here, we demonstrate a dual-split bull’s eye (DSBE) antenna, which consists of two sub-wavelength slits crossing through the center of the BE antenna with an intersection angle *θ*. The polarization dependence in transmission can be flexibly tailored by adjusting the intersection angle, following a cos^2^ (*Φ* + *θ*/2) angular dependence on polarization angle *Φ*. When *θ* = 90°, the DSBE antenna yields high and polarization-independent transmission enhancement over the entire infrared spectrum. It presents highly promising applications for polarization-insensitive photodetectors and other optoelectronic devices.

Bull’s eye (BE) structure that includes concentric grooves surrounding a sub-wavelength circular hole has potential applications in ultra-fast photodetectors, nanolithography, nanoscale imaging, optical communications and so on^1^,^2^,^3^,^4^,^5^, based on the extraordinary optical transmission (EOT) effect. As previously reported^6^,^7^, the performance of BE antenna is limited when employed in infrared photodetection due to the evanescent modes existing inside the circular hole. It has been demonstrated that the concentration ability could be successfully boosted at infrared wavelengths by exploring the idea of split bull’s eye (SBE) antenna that has a sub-wavelength slit as the central aperture^8^,^9^. As a result of breaking in rotational symmetry, the transmission enhancement of the SBE antenna exhibits a cos^2^ *Φ* angular dependence characteristic on the incident polarization angle *Φ*. It raised an issue that is deserved further improvement in the detection of unpolarized or arbitrarily polarized light.

In this work, we firstly investigate the polarization extinction ratio (PER) of transmitted light in SBE antennas for the operating wavelength from 1.3 to 4 μm. It is found that the SBE antenna has an extremely high PER, and the value depends on the structural parameters and the operating wavelength, suggesting great potential in many applications such as polarization-sensitive photodetection. To manipulate the polarization dependence of the EOT for the application involving unpolarized or arbitrarily polarized light^10^,^11^, we propose the idea to add one more sub-wavelength slit into the SBE antenna and form a dual-split bull’s eye (DSBE) antenna. The simulation results show that the transmission enhancement maintains high and follows a cos^2^ (*Φ* + *θ*/2) angular dependence on the polarization angle. Here *θ* is the intersection angle of the slits. Particularly for two orthogonal slits, the transmission enhancement is completely independent on the incident polarization state. Finally, we discuss the propagating modes and Fabry-Perot (F-P) resonances occurring in the DSBE antennas at infrared, which enable the lager transmission enhancement in contrast to BE schemes. Our results indicate that such a DSBE design will benefit significantly as a polarization-independent antenna in infrared photodetection.

## Results

### Schematic of split/dual-split bull’s eye antennas

**Figure 1:**
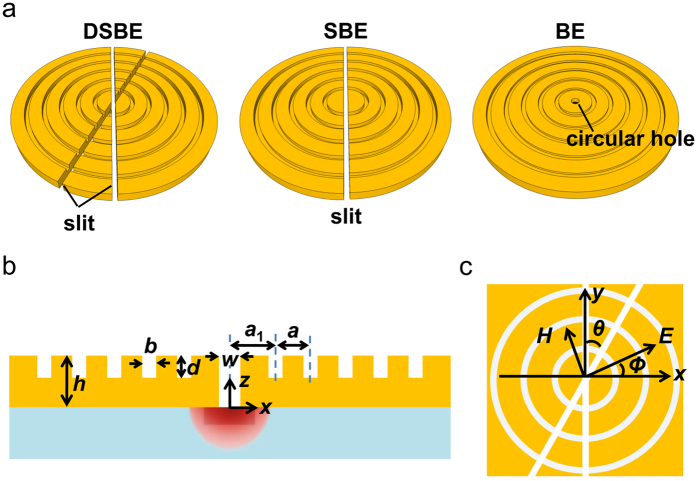
Design of DSBE, SBE and BE antennas. (**a**) 3D schematics of DSBE, SBE and BE antennas. (**b**) Cross-sectional view of the photodetector with DSBE, SBE or BE antenna. For DSBE and SBE antennas, *w* is the slit width; for BE antenna, *w* is the hole diameter. (**c**) Top view of a DSBE antenna with an intersection angle *θ* and incident polarization angle *Φ*.

Schematic diagrams for the proposed DSBE, SBE and BE antennas are sketched in Fig. 1(a). The antennas with 5 concentric grooves are constructed on a free-standing Ag film. The central aperture of the DSBE antenna consists of two crossed subwavelength slits with an intersection angle *θ* (Fig. 1(c)). Other geometrical parameters of these three antennas are identical with each other as shown in Fig. 1(b). The groove period, groove width, groove depth, film thickness, aperture size and the distance from the aperture center to the first groove are denoted as *a, b, d, h, w* and *a*_1_, respectively. All the geometrical parameters are same with our previous work from near to mid infrared^9^. Similar to ref. 12., we assume that the aperture size *w* is fixed at 0.3 μm for the intention of high-speed response in nano-photodetectors. The incident light, which is linearly polarized plane waves, impinges normally on the surface of the antennas (i.e., incident along *z*-axis). The polarization angle *Φ* is defined as the angle between the electric field and the *x*-axis (Fig. 1(c)).

Basically, the absorption of the active region beneath the antennas (see Fig. 1(b)) can be estimated by the transmission enhancement associated with the EOT effect^13^, which has been illustrated through an equation in Method part. As reported, the transmission enhancement, *η*, of the aforementioned antennas satisfied the relation^14^


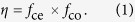


It means that the transmission enhancement *η* is determined by two independent factors: the coupling efficiency of the grooves*, f*_ce_, and the cutoff function of the central aperture, *f*_co_. Either of them can produce polarization dependence in EOT through the antennas. The example schemes can be constructed as (i) circular hole surrounded by elliptical or fan-shaped grooves^15^,^16^, wherein the factor *f*_ce_ is polarization dependent, (ii) elliptical or bowtie central apertures surrounded by circular grooves^17^,^18^,^19^, wherein *f*_co_ is polarization dependent, or (iii) polygonal apertures surrounded by polygonal grooves, wherein both *f*_ce_ and *f*_co_ are polarization dependent^20^,^21^,^22^. To further understand the impacts of light polarization, we define the PER as the ratio of the maximum to the minimum transmission enhancement for all the polarization states. As studied in ref. 9, a SBE antenna with a subwavelength slit belongs to scheme (ii) and the EOT intensity obeys a cos^2^ *Φ* angular dependence characteristic. In this case, PER can be calculated by *η*_SBE_ (*Φ* = 0°)/*η*_SBE_(*Φ* = 90°), where *η*_SBE_ (*Φ* = 0° or 90°) corresponds to the transmission enhancement of SBE antenna with *Φ* = 0° or 90° incidence.

### Polarization dependence of split bull’s eye antennas

**Figure 2:**
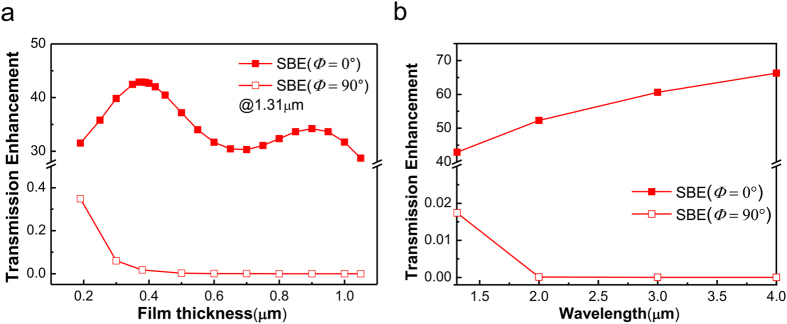
Polarization dependence of transmission enhancement in SBE antennas. (**a**) The transmission enhancement of SBE antennas versus the film thickness at *λ* = 1.31 μm with polarization angle *Φ* = 0° or 90°. (**b**) Transmission enhancement of SBE antennas at *λ* = 1.31, 2.0, 3.0, and 4.0 μm with polarization angle *Φ* = 0° or 90°. The corresponding film thicknesses *h* = 0.38, 0.64, 1.07, and 1.48 μm, respectively.

Figure 2(a) shows *η*_SBE_ (*Φ* = 0°) and *η*_SBE_ (*Φ* = 90°) of SBE antennas with respect to the metal thickness when *λ* = 1.31 μm. The groove period *a* is fixed at 1.29 μm to ensure strong coupling of surface plasmons in accordance with Bragg condition. The value of *η*_SBE_ (*Φ* = 0°) is extremely high and varies periodically with the growing film thickness. The maxima correspond to F-P resonances caused by the reflection of the waveguide modes in the subwavelength slit^9^. The value of *η*_SBE_ (*Φ* = 90°) is quite lower (<1) and goes down exponentially with film thickness increasing, which can be understood from the approximate formula of electric field in a single sub-wavelength slit with *Φ* = 90° incidence^23^


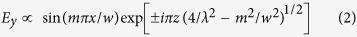


here *m* is a non-zero integer. When *λ* > 2*w*, all the modes in the slit are evanescent and the EM intensity follows an exponential decay with film thickness, resulting in a rapid increase in PER. Table 1 lists PER values at *h* = 0.38 and 0.9 μm, corresponding to the 1st or 2nd-order F-P resonances at *λ* = 1.31 μm. It is clear that the SBE antenna with a narrower slit (*w* = 0.1 μm) has a larger PER, which is mainly attributed to two reasons. First of all, the SBE antenna with a narrower central slit will get a higher *η*_SBE_ (*Φ* = 0°) as previously reported in ref. 9. Secondly, a smaller *w* will lead to a lower *η*_SBE_ (*Φ* = 90°) due to a larger imaginary part of the propagation constant according to equation (2). In addition, the value of PER will be larger when towards longer wavelengths. It has been studied in details that the value of *η*_SBE_ (*Φ* = 0°) monotonously increases when the wavelength extends from near to mid-infrared (Fig. 2(b)). The reduction of *η*_SBE_ (*Φ* = 90°) is raised by the larger imaginary part of the propagation constant (equation (2)) as well as the larger film thickness *h*, as it should be increased to support F-P resonances for the case of *Φ* = 0° incidence at longer wavelengths^9^. As a result, a very high PER of 89 dB is achieved at *λ* = 4 μm due to the large contrast between *η*_SBE_ (*Φ* = 0°) and *η*_SBE_ (*Φ* = 90°).

### Polarization tunability of transmission enhancement in dual-split bull’s eye antennas

**Figure 3:**
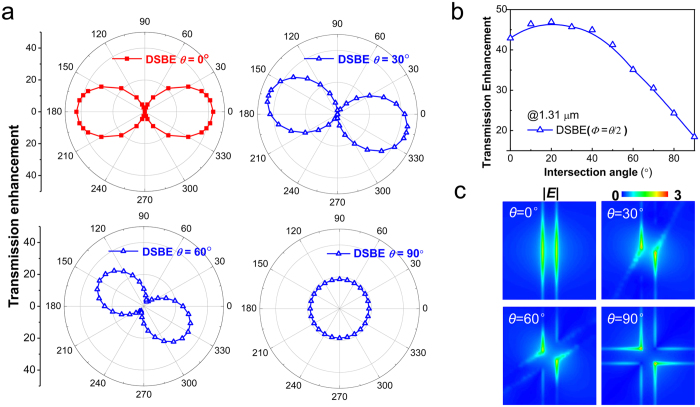
Polarization-tunable transmission enhancement through DSBE antennas. (**a**) The angular distribution of transmission enhancement of the DSBE antennas with different intersection angles in polar coordinate at *λ* = 1.31 μm. (**b**) The maximum value of *η*_DSBE_ with respect to the intersection angles. (**c**) The normalized and time-averaged |***E***| intensity distributions around the center of the antennas on the *z* = 0 plane at *λ* = 1.31 μm with *Φ* = −*θ*/*2*.

In order to overcome the issue of polarization dependence caused by the symmetry breaking, we introduce an additional sub-wavelength slit into the SBE antenna to form a DSBE antenna. The polarization dependence in EOT can be modulated by tuning the intersection angle *θ* as shown in Fig. 3(a) with *h* = 0.38 μm. Specially, the case of *θ* = 0° corresponds to a SBE antenna. It is found that the transmission enhancement of the DSBE antenna, *η*_DSBE_, obeys a cos^2^ (*Φ* + *θ*/2) angular dependence characteristic, which can be roughly estimated by treating the DSBE structure as a superposition of two independent SBE antennas. As mentioned above, 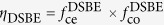, here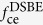 is polarization independent. Thus, the polarization dependence of a DSBE antenna is mainly determined by its cut-off function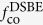, which can be written as


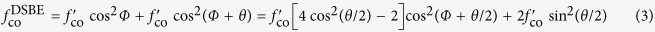


where each SBE antenna satisfies 
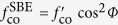
 9, 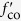 is the corresponding maximum cutoff function of each SBE antenna. Figure 3(a) shows the simulated angular distribution of transmission enhancement in polar coordinate for DSBE antennas with different intersection angles *θ*. It is found that *η*_DSBE_ reaches its maximum at *Φ* = −*θ*/2 or *π* − *θ*/2, which is in good agreement with equation (3). In particular, when the two crossed slits are orthogonal to each other, i.e., *θ* = 90°, the value of 
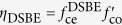
 is independent on the polarization angle *Φ*. It is concluded that, in such a way, the polarization properties of our DSBE structure can be effectively modulated, including the transmitted field intensity and polarization state.

As depicted in Fig. 3(b), if sweeping the intersection angles *θ* from 0 to 90°, the maximum value of *η*_DSBE_ simulated by finite-difference time-domain (FDTD) method will increase to a saturation point at ~20° and then decrease. Such a feature cannot be precisely described by the superposition model equation (3), in which the maximum value of *η*_DSBE_ (i.e., *η*_DSBE_ (*Φ* = −*θ*/*2* or *π*−*θ*/2)) equals 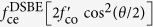, obeys a monotonous decrease function. In Fig. 3(c), we plot the normalized (with respect to the incident light) and time-averaged |***E***| intensity distributions around the center of the antennas on the *z* = 0 plane when *Φ* = −*θ*/2. It is found that the surface charge oscillations, i.e., the so-called localized surface plasmon (LSP) resonances, take place at the sharp corner of the crossed slits when the intersection angle *θ* > 0°. The induced strong near-filed enhancement makes contribution to the cut-off functions^13^, which has not been involved in superposition model. When *θ* increases from 0°, the value of 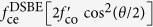 starts to decease slowly. In this case, the LSP resonances will slightly enhance the maximum value of *η*_DSBE_. When the angle *θ* larger than a characteristic point (~20°), the value of 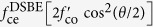 decreases dramatically, and the effect of LSP resonances can thus be negligible. Therefore, the corresponding maximum value of *η*_DSBE_is reduced with *θ* increasing up to 90°.

**Figure 4:**
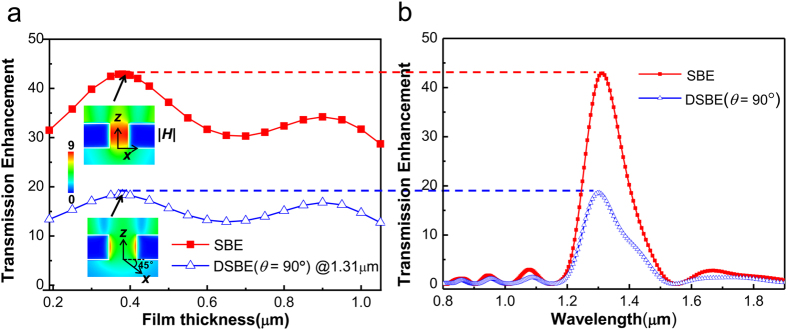
Analysis on EOT properties in DSBE antennas. (**a**) Transmission enhancement of DSBE (*θ* = 90°) and SBE antennas with respect to the film thickness *h* at *λ* = 1.31 μm. The insert shows the cross-sectional view of the normalized and time-averaged |***H***| intensity distributions for SBE and DSBE (*θ* = 90°) antennas at the F-P resonant peaks of 1.31 μm with polarization angle *Φ* = −*θ*/*2*. Each mapping plane is perpendicular to the magnetic field vector of their individual incident light. (**b**) Transmission enhancement spectra of SBE antennas and DSBE (*θ* = 90°) at *h* = 0.38 μm with polarization angle *Φ* = −*θ*/*2*.

To further understand the EOT behaviors of the polarization-independent DSBE antenna (i.e., *θ* = 90°), we investigate how the transmission enhancement depends on the metal thickness *h* at *λ* = 1.31 μm (Fig. 4(a)). With *h* increasing, the transmission enhancement of DSBE (*θ* = 90°) antenna shows similar periodicity with SBE antenna (under *Φ* = 0° incidence), which confirms the existence of propagating modes in the central aperture. In addition, according to F-P resonance condition, the F-P resonance modes will not shift in the DSBE (*θ* = 90°) antenna, as the metal thicknesses are unchanged when introducing the second slit into the SBE antenna. The inserts shows the cross-sectional distribution of the normalized (with respect to the incident light) and time-averaged magnetic field |***H***| for SBE and DSBE (*θ* = 90°) antennas with *h* = 0.38 μm, *Φ* = −*θ*/2, and *λ* = 1.31 μm. One can see that the magnetic field is confined to the gaps with the maximum field occurring along slit walls, which is due to the existence of waveguide mode and its F-P resonance^9^. As explained in refs 24, 25, 26, when the EM energy transmits through the central slit as a charge-density wave, surface currents will be formed on the slit walls and thus the magnetic field is especially strong there. As depicted in Fig. 4(b) with *h* = 0.38 μm, the transmission peaks associated with the 1st-order F-P resonance occurs at *λ* = 1.31 μm in both structures.

## Discussion

**Figure 5:**
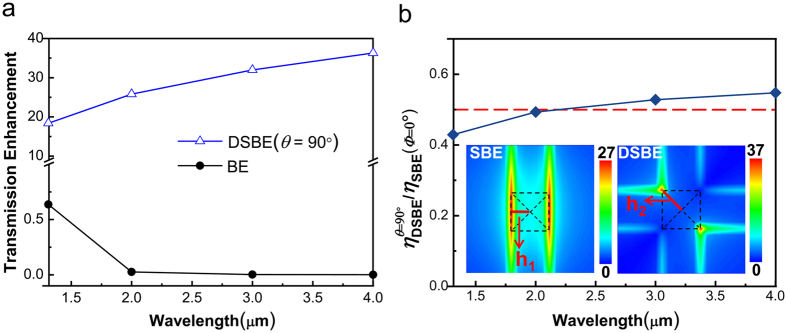
Comparison of transmission enhancement between DSBE antennas and BE/SBE antennas. (**a**) Transmission enhancement of DSBE (*θ* = 90°) and BE antennas at *λ* = 1.31, 2.0, 3.0, and 4.0 μm, and the corresponding film thicknesses *h* = 0.38, 0.64, 1.07, and 1.48 μm, respectively. (**b**) The ratio of 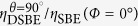 from 1.31 to 4 μm. The red dashed line corresponds to the value of 0.5. The inserts are the normalized and time-averaged |***E***| intensity distributions of SBE and DSBE antennas on the *z* = 0 plane at *λ* = 1.31 μm with *Φ* = −*θ*/*2*.

On the basis of the above analysis, the operating wavelength of DSBE (*θ* = 90°) antenna can be easily extended to mid-infrared if using the same geometrical parameters of SBE antennas. As shown in Fig. 5(a), in spite of the incident polarization, the transmission enhancement of the DSBE (*θ* = 90°) antenna increases monotonically with the operating wavelength and is about 6 orders of magnitude higher than the traditional polarization-independent BE antenna at *λ* = 4 μm. On the contrary, the transmission enhancement of the BE antenna exponentially decreases with the operating wavelength increasing. The DSBE (*θ* = 90°) antenna is therefore the prior of choice for nano-photodetectors with unpolarized or arbitrarily polarized light incidence at infrared wavelengths.

As seen from Fig. 4, the transmission enhancement of DSBE (*θ* = 90°) antennas will be reduced in half when compared to SBE antenna, which also can be evaluated by equation (3). However, the ratio of 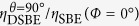 is actually dependent upon the working wavelengths if considering the different optical modes existing in the antennas. As shown in Fig. 5(b), the simulated 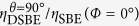 based on FDTD method is greater than 0.5 for *λ* > 2 μm and less than 0.5 for *λ* < 2 μm, which can be understood in terms of the cooperative contribution of concentric grooves and central apertures. Based on equation (1), the ratio of 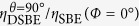 can be expressed as 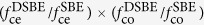. Here 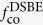 and 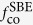 are cut-off functions that are related to *maximum near-fields* distributed at the edges (for SBE) or surrounding the sharp corner of the central aperture (for DSBE) as shown in the inset of Fig. 5(b). As discussed above, the factor 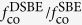 would be slightly larger than 0.5 if considering the presence of LSP modes as an additional term to the superposition model equation (3).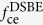 or 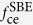 is coupling efficiency, which is determined by the concentric grooves and forms a concentration area centered at the antenna center with a gradient. Since the *maximum near-fields* in the DSBE antenna is farther away from the *concentration area* center than the SBE structure (*h*_2_ > *h*_1_ in Fig. 5(b)), we should have 
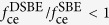
. Actually, the factor of 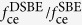 is observed with wavelength dependence. When *λ* > 2 μm, the wavelength of SPP waves (*λ*_SPP_) induced by the grooves is much longer than the size of the central aperture, i.e., (*h*_2_ − *h*_1_) ≪ *λ*_SPP_. In this case, the concentration area has a gentle gradient and we have the ratio of 
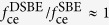
. It explains why the ratio of 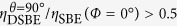 for *λ* > 2 μm as shown in Fig. 5(b). When *λ* < 2 μm, although we have 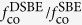 slightly larger than 0.5, the ratio of 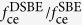 will be rapidly reduced when the wavelength decreases, since the gradient of the concentration area becomes steeper for a shorter *λ*_SPP_, which finally results in 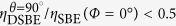.

In summary, we have studied the polarization properties of DSBE antennas from near to mid-infrared wavelengths. The polarization dependence in the EOT can be flexibly controlled by adjusting the intersection angle of the slits. When *θ* = 0°, the antenna (i.e., SBE antenna) is strong polarization dependent. The large PER can be enhanced by using a narrower sub-wavelength slit or extending the operating wavelength to mid-infrared. The maximum PER = 89 dB is achieved at *λ* = 4 μm, exhibiting excellent ability for polarization-sensitive photodetectors that only sense the signal intensity. When *θ* = 90°, the transmission enhancement of the antenna is completely polarization independent. Moreover, DSBE antennas can maintain the EOT behaviors of SBE antennas. For instance, when *λ* = 4 μm, the transmission enhancement of the DSBE (*θ* = 90°) antenna is 6 orders of magnitude higher than the traditional polarization-insensitive BE antenna. This may offer the possibility to unyoke the limitation of SBE antennas in polarization independence and facilitate the applications of optical antennas in optical communication devices.

## Methods

### Numerical simulations

Three-dimensional FDTD simulations were performed using a commercially software package (Rsoft FullWAVE) with perfectly matched layer (PML) boundary conditions. The transmission enhancement is defined as the ratio of the integrated *z*-component of the Poynting vector ***S*** (***S*** = ½Re(***E*** × H^*^)) over the output and input apertures. The integral domains of DSBE and SBE antennas are 0.3 μm × 0.3 μm square areas at the center of the antennas. The permittivity of Ag follows the Lorentz-Drude model at all operating wavelengths. A non-uniform orthogonal mesh grid is used to reduce the computational costs. The mesh size at the material interfaces is set to be 5 nm which is much smaller than the element sizes and the operating wavelength, and the calculations converged well.

## Additional Information

**How to cite this article**: Yang, M. *et al*. Polarization-independent split bull’s eye antennas for infrared nano-photodetectors. *Sci. Rep.* **6**, 39106; doi: 10.1038/srep39106 (2016).

**Publisher's note:** Springer Nature remains neutral with regard to jurisdictional claims in published maps and institutional affiliations.

## Figures and Tables

**Table 1:** *η*_SBE_ (*Φ* = 0° or *Φ* = 90°) and the corresponding PER with different geometrical parameters.

SBE	*h* = 0.38, *w* = 0.3 μm	*h* = 0.9, *w* = 0.3 μm	*h* = 0.38, *w* = 0.1 μm
*η*_SBE_ (*Φ* = 0)	43	34	105
*η*_SBE_ (*Φ* = 90)	1.7E-2	5.6E-6	1.6E-6
PER	34 dB	68 dB	78 dB
